# Correction: The thermodynamics and kinetics of depolymerization: what makes vinyl monomer regeneration feasible?

**DOI:** 10.1039/d4sc90022g

**Published:** 2024-01-30

**Authors:** Victoria Lohmann, Glen R. Jones, Nghia P. Truong, Athina Anastasaki

**Affiliations:** a Laboratory of Polymeric Materials, Department of Materials ETH Zürich, Vladimir-Prelog-Weg 5 8093 Zürich Switzerland athina.anastasaki@mat.ethz.ch; b Monash Institute of Pharmaceutical Sciences, Monash University 399 Royal Parade Parkville VIC 3152 Australia

## Abstract

Correction for ‘The thermodynamics and kinetics of depolymerization: what makes vinyl monomer regeneration feasible?’ by Victoria Lohmann *et al.*, *Chem. Sci.*, 2024, **15**, 832–853, https://doi.org/10.1039/D3SC05143A.

The authors regret the mislabelling of Fig. 4 as Fig. 5 (and *vice versa*) and Fig. 15 as Fig. 16 (and *vice versa*), resulting in discrepancies between text references and the figures. The correctly labelled figures are shown below:

**Fig. 4:**
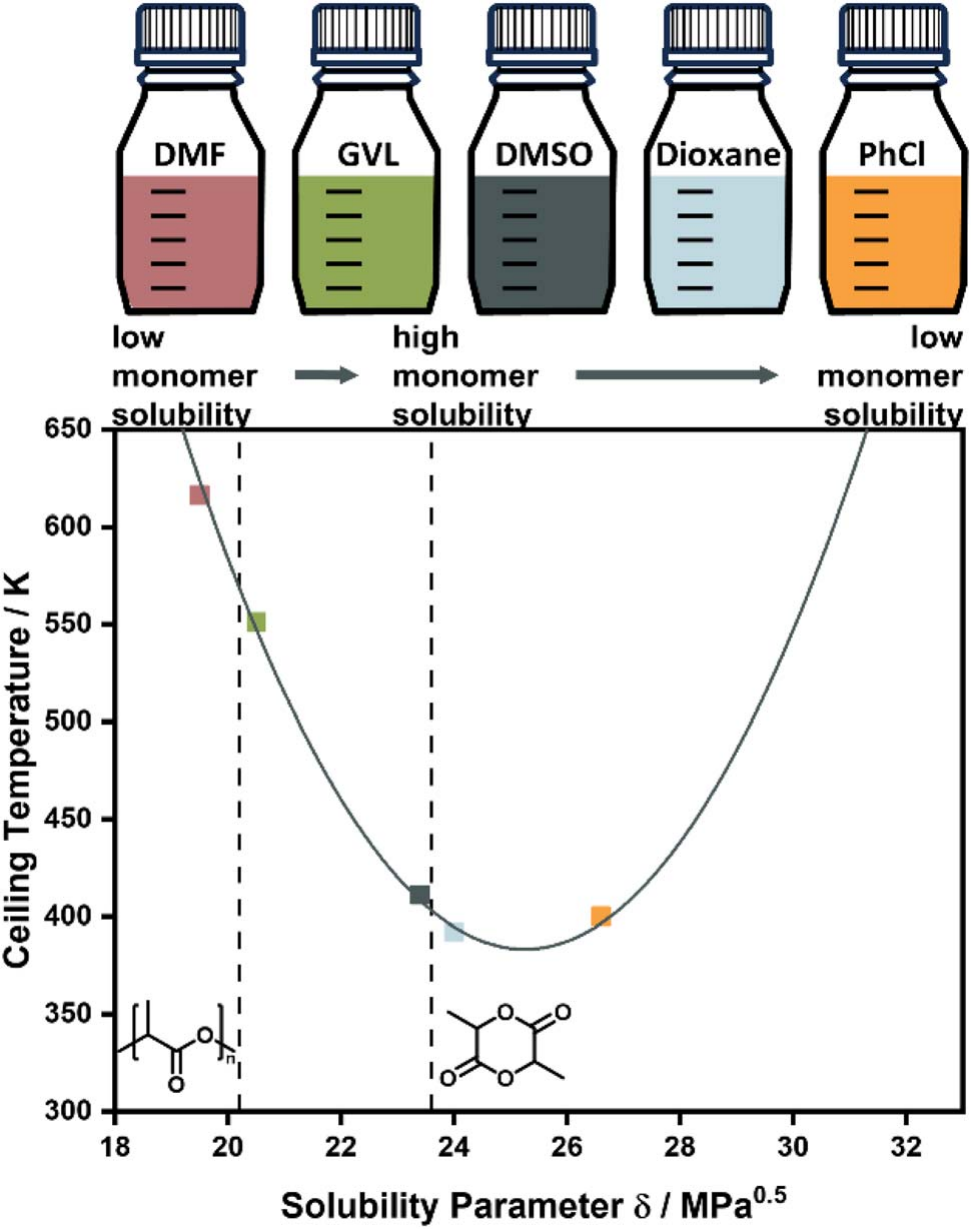
Changing the ceiling temperature through choice of solvent. The smaller the solubility difference between monomer and solvent, the lower the ceiling temperature. Adapted from ref. 71 “Like Recycles Like”: Selective Ring-Closing Depolymerization of Poly(l-Lactic Acid) to l-Lactide, L. Cederholm, J. Wohlert, P. Olsén, M. Hakkarainen and K. Odelius, *Angew. Chem. Int. Ed.*, **61**, 33. Copyright © 2022 the authors.

**Fig. 5:**
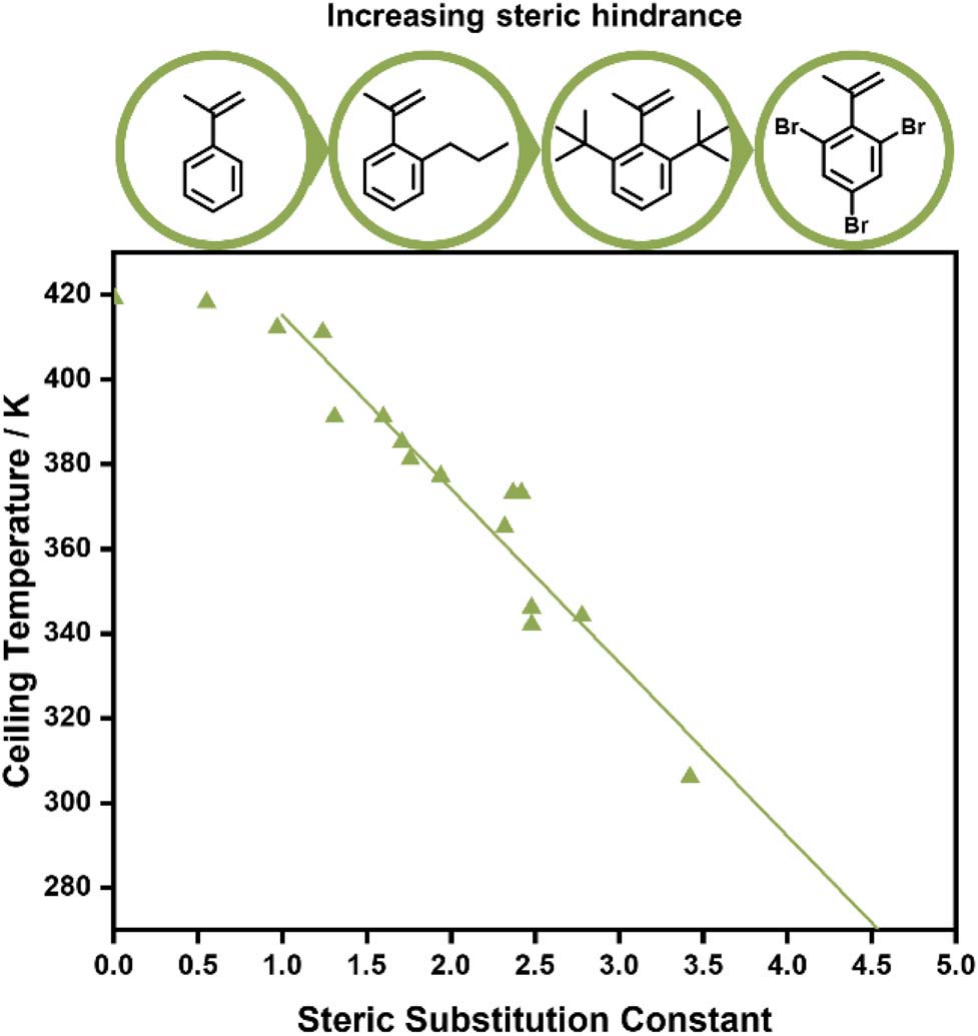
The effect of phenyl methacrylate’s substituents on the ceiling temperature, the larger the substituent the lower the ceiling temperature of the polymerization. Graph reprinted from ref. 90 B. Yamada, T. Tanaka and T. Otsu, Correlations of ceiling temperature and reactivity with bulkiness of *ortho*-substituent in radical polymerization of phenyl methacrylate, *Eur. Polym. J.*, **25**, 2, 117–120, copyright 1989, with permission from Elsevier.

**Fig. 15:**
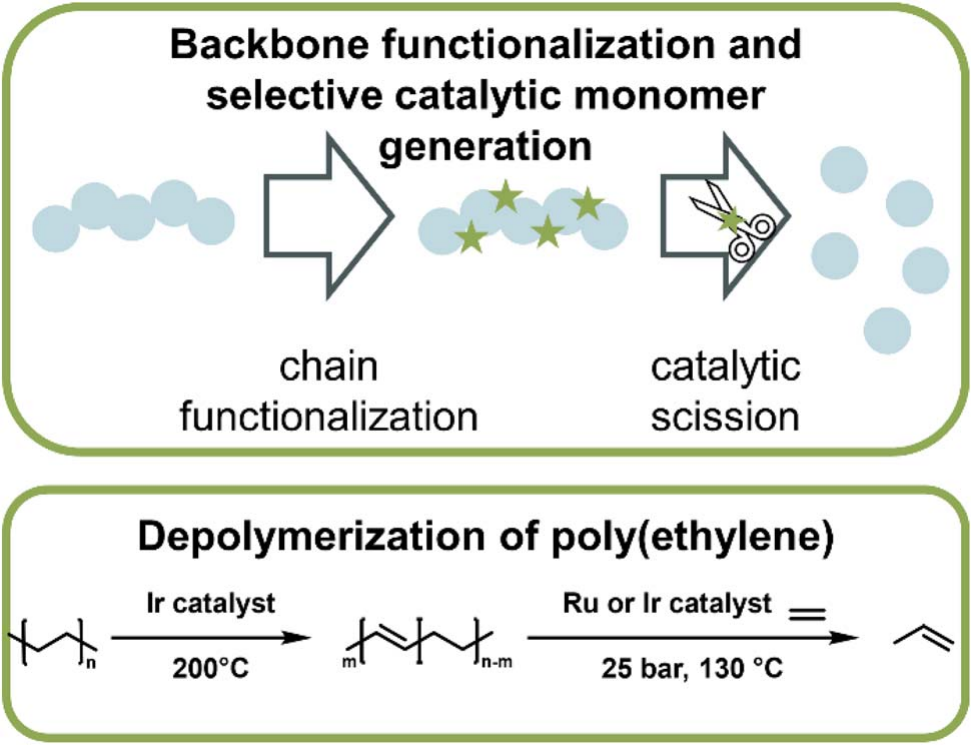
Schematic overview of catalytic monomer regeneration from previously functionalized polymer backbones with selective catalytic sites (top). Example of depolymerization of dehydrogenated polyethylene with ruthenium and iridium catalysts (bottom) as described in ref. 162.

**Fig. 16:**
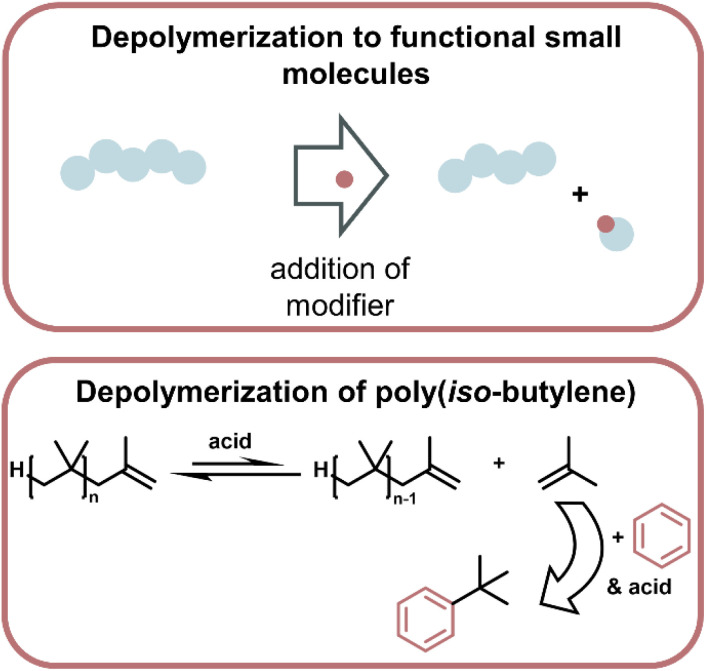
Schematic overview of depolymerization driven by modification of monomer to make functional small molecules, which then thermodynamically drives the depolymerization (top). Such a system was applied to the depolymerization of poly(iso-butylene) which was driven by the highly enthalpically favoured Friedl–Craft type reaction of the monomer with benzene in the presence of strong acid (bottom) as described in ref. 169.

The Royal Society of Chemistry apologises for these errors and any consequent inconvenience to authors and readers.

## Supplementary Material

